# Why should medical editors CARE about case reports?

**DOI:** 10.3325/cmj.2013.54.507

**Published:** 2013-12

**Authors:** Hrvoje Barić, Lidija Andrijašević

**Affiliations:** *Croatian Medical Journal*, Zagreb, Croatia

“Always note and record the unusual…Publish it.” (1) William Osler

While contemplating the evolution of medical publishing, one might be tempted to think about it in terms of Recapitulation theory. This theory claims that the development of an individual animal from embryo to adult resembles the stages that that species has undergone in the course of its evolution. Therefore, it might be conjectured that the stages of scientific production in an individual clinician`s career follow similar progression as the history of medical publishing. In other words, the first medical articles were written in a form that resembled today’s case reports, while more complex forms, such as randomized controlled trials or reviews and meta-analyses, appeared much later. Similarly, individual clinicians, through their career, climb the steep “level-of-evidence“ pyramid; during the first clinical steps, they write case reports, striving towards taking part in reporting clinical trials, and, later as they grow proficient in their field of interest write reviews and meta-analyses. Even though case reports are indispensable for medical progress since they bring attention to novel entities, in the evidence based era of impact factors and citations, they are often considered to be less valuable, due to their low citation rates (2). However, case reports have not only changed and grown more complex in their form, but continue to report on a wide range of topics other than direct clinical experience. Similarly, the role of case reports has outgrown its primary purpose. In its beginnings, case reports were intended to identify clinical novelty, afterwards authors used them as a means to alert about adverse reactions and highlight innovations, while today they play a significant role in medical education and help emphasize ethical predicaments (3,4).

## HISTORICAL PERSPECTIVE

Throughout the history of medicine, case reports have played a major role, some of them subsequently becoming highly cited works that often represent cornerstone publications in their fields. Hippocrates, the father of modern medicine, reported his findings in what would today be considered anecdotal form (5). Sigmund Freud used case reports to describe the cases of his patients, which are now considered famous and mark the beginning of psychoanalysis. Four weeks after having performed the first human heart transplantation on December 3, 1967, Christian Barnaard published a case report in the *South African Medical Journal* (6). Case reports once again proved to be crucial in 1981, when a series of such publications reporting unusual infections and neoplasms led to the discovery of a new syndrome, AIDS (7-9). A recently published case series, which reported on a novel therapeutic option for Shiga-toxin producing *Escherichia coli –* hemolytic-uremic syndrome, led to advancement in the treatment protocol for refractory cases (10,11). Although this example clearly shows that case reports are still an invaluable means of communicating medical novelty, from 1970s the proportion of published cases has steadily declined (2). The 1990s saw the re-emergence of case reports. Several eminent journals made a turn toward a “case report friendly policy,” while editorials and commentaries indicated a rise of interest in case reports (2,12,13). Finally, the 2000s witnessed the appearance of first exclusively case report journals (14).

## THE CARE GUIDELINES

Recently, a group of eminent experts involved in case report writing and editing have addressed the issue of standardizing case reports (15). The authors pointed out that there were guidelines for other publication types that facilitated standardization. For example, when publishing meta-analyses, authors adhere to PRISMA guidelines, and when publishing randomized controlled trials they adhere to CONSORT guidelines (16,17). The list goes on and encompasses mostly all types of publications and, until recently, did not include international reporting case report guidelines. However, in September 2013, CARE (CAse REport) guidelines were presented and published in several journals (18).

## CASE REPORT COMEBACK

Why should we care about case reports? Being on the bottom of the pyramid, case reports are often neglected by both publishers and readers (perhaps even authors?). However, their educational role is often overlooked. They present a case of a single individual, a unique patient, the kind one has in mind when studying a disease and projecting symptoms into an imaginary patient; and every patient, no matter how “ordinary” his or her problem, has something to teach us. In this respect, case reports may be one of the ways to recover some of the “lost humanity of medicine” (19). Higher level-of-evidence articles are more refined and highly scientific, but case reports, due to lack of guidelines and difficulties in getting them published often demand from the author writing and presentation skills that more resemble art than science. Moreover, they represent an “atavism” seen from the epistemological point of view; among the multitude of “conjectures and refutations” case reports are “the last resort of inductionism” (20,21).

An analysis of the *CMJ*’s impact factor (IF) structure for 2003 (22) showed that case reports indeed contributed the least to the IF (relative IF only 0.19, compared to 0.91 for original articles and 0.63 for reviews) (22). The biggest contribution was provided by Editorials and Forum (22). Interestingly, journals with the highest IFs have been using results of such citation analyses for years now – in *Nature* 63% of published papers were categorized as non-research items (editorials, letters, etc.) and in *New England Journal of Medicine* 81% items (23). The same paper reports on how certain papers classified as non-research items by journals actually contained original research data.

It is clear to *CMJ* editors, as it obviously is to editors of many larger journals with greater influence, that it would be wiser to publish reviews, original research, letters, correspondence, than case reports. Therefore, many editors now ask authors to restructure case reports (not only case reports, even original research!) into Letters to Editors. This way the case report gets published, but might get “crippled” in the restructuring process. However, they can get citations, without influencing the denominator when calculating IF.

Is this really the way we are headed? And are we truly putting IF and journal ranking in the first place, and disregarding potential benefits and contribution a case report or a full research-article can give to scientific community? Though we also are struggling to survive in a community where IF has a divine status, and its oscillations directly affect the life of a journal and its editors, we support the “case report comeback.” In a constant struggle to survive and increase/maintain our IF, we will not forget that we are a small journal striving toward quality and excellence before anything else (including bibliometric parameters!). A good article will always get published – even if it might increase our denominator. As stated in our Guidelines for Authors, case reports still have a low priority in the *CMJ*. However, this does not mean they cannot get published. Hereby, we encourage our authors to read and apply new CARE Guidelines – they can help your case report get included into the “who **CARE**s if it increases the denominator” category.

We invite you to read our three new case reports (24-26) and dare you to cite them!
